# Different Effects of Hypoxia on Mental Rotation of Normal and Mirrored Letters: Evidence from the Rotation-Related Negativity

**DOI:** 10.1371/journal.pone.0154479

**Published:** 2016-05-04

**Authors:** Qingguo Ma, Linfeng Hu, Jiaojie Li, Yue Hu, Ling Xia, Xiaojian Chen, Wendong Hu

**Affiliations:** 1 Institute of Neural Management Sciences, Zhejiang University of Technology, Hangzhou, China; 2 School of Management, Zhejiang University, Hangzhou, China; 3 Neuromanagement Lab, Zhejiang University, Hangzhou, China; 4 Hangzhou Aviation Medicine Assessment and Training Center of Air Force, Hangzhou, China; 5 Faculty of Aerospace Medicine, The Fourth Military Medical University, Xi’an, China; Georgia Regents University, Medical College of Georgia, UNITED STATES

## Abstract

The present study explored the neural mechanism underlying the effect of moderate and transient hypoxic exposure on mental rotation of two-dimensional letters in both normal and mirror versions. Event-related potential data and behavioral data were acquired in the task of discrimination between normal and mirrored versions separately in conditions of normoxia (simulated sea level) and hypoxia conditions (simulated 5000 meter altitude). The behavioral results revealed no significant difference between the normoxia and hypoxia conditions both in response time and error rate. However, obvious differences between these two conditions in ERP were found. First, enlarged P300 and Rotation-related Negativity (RRN) were observed in the hypoxia condition compared to the normoxia condition only with normal letters. Second, the angle effect on the amplitude of RRN was more evident with normal letters in the hypoxia condition than that in the normoxia condition. However, this angle effect nearly disappeared with the mirrored letters in the hypoxia condition. Third, more bilateral parietal activation was observed in the hypoxia condition than the normoxia condition. These results suggested that the compensation mechanism existed in the hypoxia condition and was effective with normal letters but had little effect on the mirrored letters. This study extends the research about the hypoxic effect on spatial ability of humans by employing a mental rotation task and further provides neural evidence for this effect.

## Introduction

Hypoxia is inevitable when people live and work at a high altitude or in other hypoxic conditions. Compared to life at sea level, people at a high altitude often suffer from some cognitive deficits due to the low level of oxygen [1]. Many studies have explored the effect of hypoxia on human cognitive functions, such as spatial attention and memory, perception, language, executive function, and psychomotor process (see [2, 3] for reviews). Spatial ability is the individual ability to deal with objects in space mentally and can be measured by spatial perception, mental rotation, and spatial visualization [4–6]. Among them, mental rotation is an important way to measure the ability of individuals to mentally represent and transform spatial objects that rotate at various angles or are in their mirrored-reversed version [7–11], which are the cores of spatial intelligence [12]. As spatial ability plays an important role in daily life, it is necessary to explore the influence of hypoxia on this ability. Previous studies have explored the hypoxic effect on some visuo-spatial abilities such as the spatial working memory [13] and spatial attention [1]. However, to our knowledge, few studies have investigated the influence of hypoxia on mental rotation, especially at the neural level. It is important to know this mechanism because people in hypoxic environments may suffer both life and property loss if this hypoxia-induced deficit in spatial ability exists. This issue is also relevant for the aviation and driving.

Mental rotation was first discussed by Shepard and Metzler [7]. In their study, the participants were instructed to judge whether the presented pair of two three-dimensional solid cubes were congruent in shape [7]. They found the typical result that the response time increased linearly with the angular discrepancy between the two cubes both in and out of the picture plane. Shepard and colleagues explained that mental rotation is a psychological process in which people form a mental representation (analog) of an object and then rotate it to the upright position or pre-learned orientation to eliminate the angular difference to make a judgment of the object (e.g., a parity judgment, left-right hands judgment) [14, 15]. Following Shepard and Metzler’s work, further mechanisms of mental rotation have been explored by both behavioral and neural studies by applying modified tasks with various stimuli [8–10, 16, 17]. These stimuli can be broadly categorized into external objects (e.g., letters, digits), human shapes (e.g., hands, other body parts) and complex scenes (e.g., landscapes, table scenes) [18]. In current study, we mainly focused on the mental rotation of external objects in the modality of two-dimensional letters as previous studies did [9, 16, 19, 20]. Normal-mirror parity judgment task with letters is a classic experimental paradigm since Cooper and Shepard (1973) and frequently applied to examine the cognitive mechanisms underlying the mental rotation [21]. It has been proposed that in order to make a decision about whether the letter is mirrored or normal, it must be rotated to its upright position [19, 20]. In other words, mental rotation is necessary for the parity judgment. Therefore, longer response time is needed with larger-rotated letters. However, this orientation-depended function of response time in parity judgment shows a curve trend [19, 20, 22], which deviates from classic linear function found in Shepard and Metzler’s work [7]. This phenomenon might result from a mixture of rotation and nonrotation processes [20, 22]. That is, small-rotated stimuli from upright would require a less proportion of trials that use mental rotation than large-rotated stimuli do [19, 22], resulting in the indifference of response time between small angular discrepancy and upright [9, 20]. Previous mental rotation studies mainly focused on the effect of stimuli angle, but few of them specified the different mechanisms between mental rotations of normal and mirrored stimuli (i.e. the effect of stimuli version). Identification and rotation of mirrored objects are also important in our daily life, such as in the conditions of driving and aviation. Thus, recent studies have begun to concentrate on the issue of how the normal and mirrored stimuli are processed differently [9, 10, 19, 23]. A systematic behavioral result is that judgment of mirrored stimuli takes a longer time than normal ones do [9, 19, 20]. Hamm et al. attributed this delay in decision time of mirrored stimuli to an extra mental rotation out of the picture plane following planar rotation to the upright position, namely the flip [19]. This flip process is similar to the mental rotation in the plane since it activates similar brain areas [23]. Previous studies have found mental rotation of three-dimensional stimuli requires more time than two-dimensional ones, suggesting higher difficulty in mental rotation in the three-dimensional space [24–26]. Therefore, the mental rotation of mirrored letters is expected more effortful than normal ones due to the additional flip rotation in the three-dimensional space [10].

In addition to the behavioral results, researchers have also found neurophysiological evidence of mental rotation. According to previous ERP studies, a classical neurophysiological indicator of mental rotation is rotation-related negativity (RRN), which is primarily distributed over the parietal regions [16, 17, 27]. This component occurs between 300 and 800 ms after the onset of stimuli and is negatively related with the discrepant angles of rotation [17, 27]. This negative trend is regarded as the overlap of a slow negativity evoked by greater angular disparities and a simultaneous P300, which is independent of angular disparity [16]. The functional relationship between this modulation of amplitude and mental rotation has been validated by several studies (see [27] for review). For instance, the angle effect of rotated stimuli on the RRN amplitude would not occur in the task that does not require the mental rotation, such as character classification [28, 29]. In normal-mirror parity judgment, normal and mirrored stimuli have the differences not only in response time but also ERP effect. It has been observed that angle effect over the RRN amplitude is delayed with mirrored stimuli relative to the normal ones, and less evident for mirrored stimuli, which are also due to the extra flip rotation [9, 19]. The posterior parietal cortex has been supported by many neuroimaging studies to involve in the mental rotation during a parity decision making [10, 21, 23, 30–32], which is consistent with the ERP findings [9, 17, 27, 33]. However, a controversy on which hemisphere (left or right) is mainly engaged in mental rotation exists [9, 34]. The dimensionality of the stimuli might account for, in part, this hemispheric difference. Roberts and Bell [35] found greater left parietal activation when performing the two-dimensional mental rotation task. However, in the complex three-dimensional task, the right hemisphere occupied a dominant. Ditunno and Mann [36] found that patients with right parietal lesions performed worse in a three-dimensional mental rotation task that was the same as Shepard and Metzler (1971)’s paradigm than those with left-sided lesions and normal healthy persons. However, Metha and Newcombe [37] found that patients with left-sided lesions suffered from impairment of mental rotation of two-dimensional geometrical shapes. Another study using Positron Emission Tomography (PET) found that greater activation of the left parietal area was elicited during a task of normal-mirror discrimination of alphanumeric stimuli requiring mental rotation [23]. This left-hemispheric dominance was also supported by an ERP studies with similar parity judgment task [9]. In contrast, some studies demonstrate bilateral activation in parietal core regions [21, 32, 38, 39].

Previous studies have found some evidence about the hypoxic effect on the mental rotation. In one study, Zhang et al. [40] found people who had lived at a high altitude (2300–4400m) for 2 years required more time for mental rotation than those who lived at sea level, and this effect was correlated with superior frontal gray matter volume. While another study did not observed the deficits in mental rotation at a simulated hypoxia condition equivalent to 4500m for less than one hour [41]. The second study suggested a compensation mechanism in the hypoxia condition. It has been verified that human would rely on several compensation mechanisms e.g., hyperventilation, tachycardia and polycythaemia, to maintain cerebral oxygen delivery in the hypoxia condition, which help neutralize the cognitive deficits to some extent [2, 42]. The inconsistent results between the above two studies might be due to the duration of exposure to hypoxia that is a critical factor determining the degree of compensation [43]. For example, Sharma et al. found visuo-spatial executive function was impaired progressively as the duration at a high altitude increased [44]. Another factor that influences the relationship between hypoxia and cognitive impairments is the task difficulty [1, 2, 41]. For instance, Wang et al. [1] found the hypoxic effect on spatial attention processing occurred only in the high perceptual load condition, resulting in the depletion of processing capacity reflected by smaller P3 amplitude in the high altitude condition. Bartholomew et al. [41] found the hypoxic exposure indeed reduced the performance of the readback tasks with high memory load only. These results suggest that due to the hypoxia, the cognitive resources might not be enough to process information in the difficult tasks.

However, the previous two studies regarding the mental rotation did not specify the paradigm applied nor cognitive processes of mental rotation. Therefore, the specific neural mechanism underlying the hypoxic effect on mental rotation still has not been clearly understood. Moreover, many studies have found that hypoxia impairs the brain regions, including the parietal lobe, which are related to mental rotation [2, 42, 45, 46]. Hence, we proposed that hypoxia might influence the process of mental rotation. In the present study, we aimed to address this issue with the classic task of discriminating normal letters from mirrored letters by using the ERP method to record EEG data during moderate and transient hypoxia exposure. In the experiment, we simulated a 5000m-altitude hypoxic environment and the exposure time to hypoxia for each participant was less than 15 minutes. This hypoxic level is found to lie within the effective compensation range of cognitive functions as reported in previous studies [2, 42, 47]. Therefore, we expected a compensation effect of hypoxia on the mental rotation might exist. Besides, since the mental rotation process of mirrored objects involves an extra flip rotation in the three-dimensional space compared to normal ones [9, 19], which might give rise to higher difficulty in processing mirrored letters [24–26], we speculated about different effects of hypoxia on these two versions of objects. Specifically, less effective compensation effect (if it existed) on the mental rotation of mirrored letters than normal ones was predicted. Given the functional relationship between rotation-related negativity and mental rotation [27], we applied the modulation of RRN to reflect neural activities responsible for the hypoxic effects on the mental rotation of normal and mirrored letters.

## Methods

### Participants

To eliminate the gender effect on mental rotation (e.g., [10, 48]), we only recruited twenty healthy male participants from the university to participate in the experiment. Data from three participants were excluded because of excessive recording artifacts or a lack of enough trials for superposition in the hypoxia condition. The remaining seventeen participants (age ranged from 20 to 25 years, mean age = 22, SD = 1.90) were tested to be right-handed according to Oldfield [49]’s handedness questionnaire and reported normal or corrected-to-normal visual acuity without any history of neurological or mental diseases. This study was approved by the Neuromanagement Laboratory ethical committee at Zhejiang University. All participants provided written informed consent before the experiment and were paid for their participation.

### Materials

**Fig 1:**
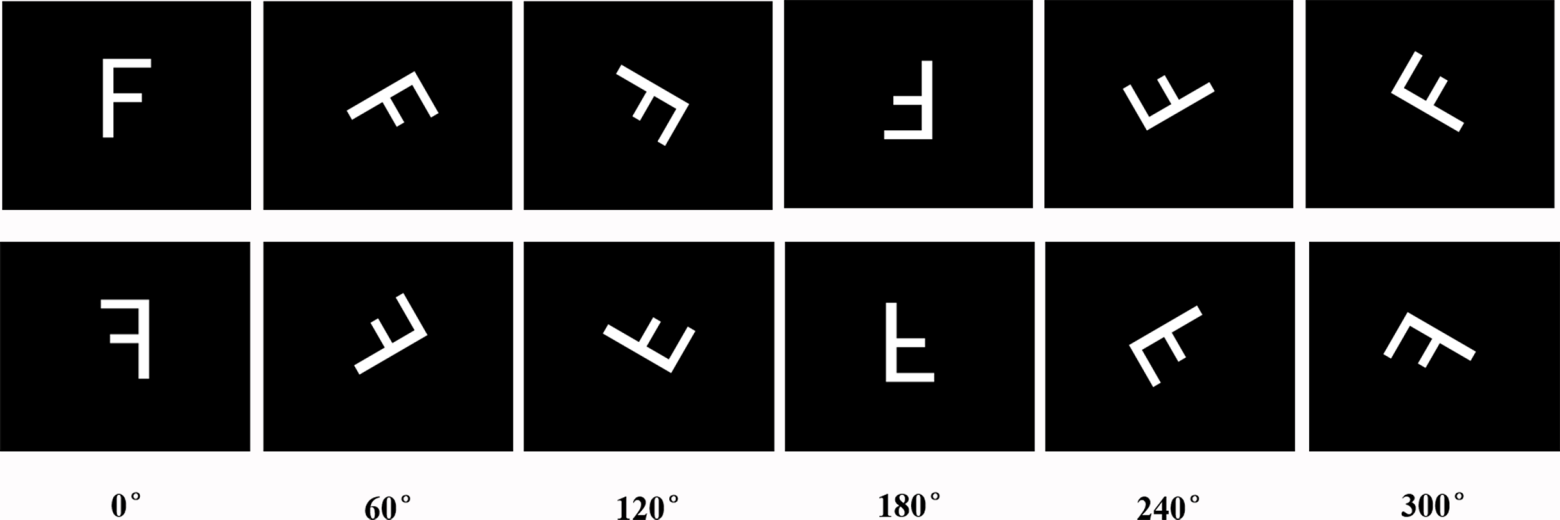
Examples of normal and mirrored letters at various angles.

The stimuli in our experiment were the six letters including F, G, L, P, Q and R adapted from previous studies [16, 19], which were presented at the angles of 0°, 60°, 120° 180°, 240° and 300° both in normal and mirror-reversed versions (see Fig 1). The letters were shown in white at the center of the computer screen with a black background. The screen was positioned 0.8 m away from the participants, resulting in a vertical visual angle of 4.58° and a horizontal visual angle of 3.05°.

### Experiment Procedure

#### Hypoxic condition

According to previous studies, there were two main methods to simulate hypoxic environment. The first is decreasing the oxygen concentration of the gas mixtures to breath, and the second is decreasing the barometric pressure which results in hypobaric hypoxia [50]. Given the convenience and low cost, the first model has been applied by many studies [51, 52]. Therefore, we chose this experimental model.

We mixed dry and clear air with nitrogen gas to produce the normoxic or hypoxic gas mixture and the concentration of oxygen was corrected by an oxygen measuring device. In the normoxia condition, the gas mixture had the same concentration of oxygen as sea level air (fraction of inspired oxygen was about 20.95%). In the hypoxia condition, the concentration of oxygen was approximately manipulated to simulate an altitude of 5000 m (fraction of inspired oxygen was about 10.5%). All the participants completed the mental rotation task in both the normoxia and hypoxia conditions. The two conditions were conducted successively with a 3-minute break. During the experiment, the participants needed to wear a mask tightly through which we transmitted the gas mixture for them to breathe, and the participants did not know which gas mixtures they were breathing in the experiment. The task in each condition did not last more than 15 minutes. Moreover, the oxygen saturation (S_a_O_2_) of each participant was monitored with a Patient Monitor iPM8 (Mindray Medical International Ltd., Shenzhen, China) during the entire experiment to ensure their safety. When the S_a_O_2_ was lower than 70% [47], we would stop the experiment immediately.

#### Mental rotation task

The experiment program was developed by E-prime software (Psychology Software Tools Inc., Pittsburgh, Pennsylvania, USA). Participants were comfortably seated in a sound-proof room facing the computer screen. They were first instructed on the task, which was to judge whether the displayed letter was normal or mirrored, and they were encouraged to respond as soon as possible and avoid errors. Then, the participants practiced to become familiar with the task and achieved at least a 90% accuracy rate.

In each condition, there was a total of 360 trials with each of the 72 factorial combinations of the following variables consisting of angles (0°, 60°, 120°,180°, 240° and 300°), version (normal and mirrored), and types of stimulus (F, G, L, P, Q and R) presented five times. The trial sequence was random. In each trial, a fixation cross was presented for a random duration between 200 and 300 ms, followed by the letter with a 2000 ms-presentation time. During this time, participants were asked to press a corresponding button with the left/right thumb on a keyboard to decide whether the letter was the normal or mirrored version as soon as possible. Finger assignment was counterbalanced across participants. Finally, a black screen occurred for 300–500 ms before the next trial.

### EEG data Acquisition

The EEG data were continuously recorded with an electrode cap containing 32 Ag/AgCl electrodes according to the standard international 10–20 system. We used the left mastoids as the reference and placed the ground electrode on the cephalic location. Vertical electrooculograms were recorded by using the two electrodes that were placed above and below the left eye, and horizontal electrooculograms were recorded with the pair of electrodes 1 cm from each eye’s lateral canthi. The EEG was recorded (band pass 0.05–100 Hz, sampling rate 500 Hz) using the Neuroscan NuAmps Amplifier (Scan 4.3.1, Neurosoft Labs, Inc. Sterling, USA), and the electrode impedance was maintained below 5kΩ throughout the experiment.

### Data analysis

#### Behavioral data analysis

**Fig 2:**
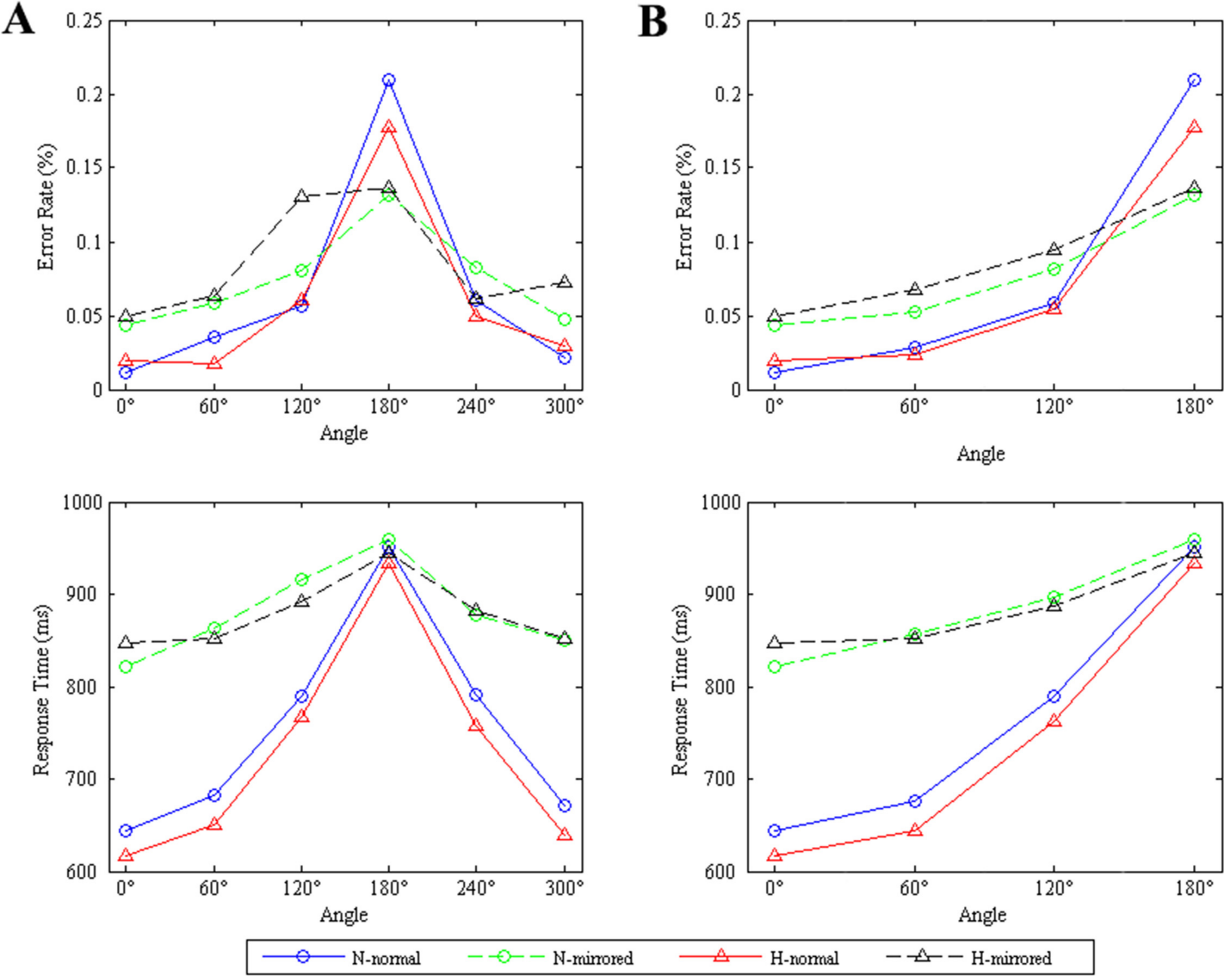
**Mean error rate (upper) and response time (bottom) as a function of all rotational angles (A) and the combined four angles (B).** The blue solid line with circle denotes the normal letters in normoxia condition, the red solid line with triangle denotes the normal letters in hypoxia condition, the green dashed line with circle denotes the mirrored letters in normoxia condition, and the black dashed line with triangle denotes the mirrored letters in hypoxia condition.

Previous studies showed that the response times and error rate of rotation were approximately symmetric for the same angular disparity clockwise and counterclockwise [9, 19, 20, 53]. After an initial analysis of the behavioral data, the symmetries about 180° were detected in our study (see Fig 2(A)). Thus, we combined the 60°-300° and 120°-240° rotations for the following analyses. Only the response times of accurate judgments were analyzed further. The behavioral data (error rate and response time) were analyzed with within-participant repeated-measure analysis of variance (ANOVA), using the hypoxic level (normoxia vs. hypoxia), version (normal vs. mirrored) and angle (0°, 60°, 120° and 180°) as within-participant factors. The Greenhouse-Geisser correction was applied for nonsphericity if appropriate. Additionally, we used the Bonferroni correction for multiple comparisons.

#### ERP data analysis

Offline preprocessing of the EEG data was conducted by the software Scan 4.5 (Compumedics NeuroScan Inc., Herndon, Virginia, USA). The ocular artifacts were corrected, and the EEG recordings were segmented into epochs of 1000 ms periods from 200 ms before the stimuli onset to 800 ms after the onset. The 200 ms pre-stimulus was set as the baseline. We excluded the trials with artifact (voltage exceeding ±80 μV) and incorrect judgments. The EEGs were digitally filtered with a low-pass filter at 30 Hz (24 dB/Octave). Finally, the data were averaged separately for each angle and version in normoxia and hypoxia conditions, respectively.

Based on visual inspection and previous studies [9, 17], the time window of rotation-related negativity was chosen as 400–500 ms to analyze the mean amplitude and latency. According to previous studies, the RRN related to the mental rotation was mainly distributed over the parietal regions containing the electrodes P3, Pz and P4 [17]. Thus, we selected these three electrodes to analyze the RRN amplitude and latency. Similar to the analysis of the behavioral data, we also used the within-participant repeated-measure analysis of variance (ANOVA) to analyze the mean amplitude and latency of RRN with hypoxic level (normoxia vs. hypoxia), version (normal vs. mirrored), angle (0°, 60°, 120° and 180°) and electrode (P3, Pz, P4) as within-participant factors. The Greenhouse-Geisser correction and Bonferroni correction were applied if appropriate.

## Results

### Behavioral results

In the error rate analysis, the main effect of angle was significant (F_(3, 48)_ = 29.719, p<0.001, ε = 0.408), but no such effects of hypoxic level and version were found (F_(1, 16)_ = 0.852, p = 0.370; F_(1, 16)_ = 0.063, p = 0.805, respectively). The error rate increased with larger angular disparity from the upright position (see Fig 2(B)). Moreover, only the interaction between angle and version was significant (F_(3, 48)_ = 6.555, p = 0.011, ε = 0.458). The angle simple effects revealed significant for both normal and mirrored letters (F_(3, 48)_ = 23.189, p<0.001, ε = 0.370; F_(3, 48)_ = 13.570, p<0.001, ε = 0.602, respectively). Specifically, there were significant linear (F_(1, 16)_ = 28.215, p<0.001), quadratic (F_(1, 16)_ = 15.838, p<0.001) and cubic (F_(1, 16)_ = 6.258, p = 0.024) trends for normal letters, whereas there was only a significant linear trend (F_(1, 16)_ = 19.681, p<0.001) for mirrored letters. Additionally, the paired contrast results showed that with normal letters, the difference between angles was significant (all adjusted-p <0.005) except for that between 0° and 60° (p = 0.516), whereas there were no differences between 0°–60° and between 60°–120° (all adjusted-p >0.05) with mirrored letters. For the version effect at each angle, we found that only at 0° and 60°, the effect of version was significant (F_(1, 16)_ = 5.427, p = 0.033; F_(1, 16)_ = 7.513, p = 0.015, respectively). The error rates of mirrored letters were larger than those of normal letters.

In the analysis of response time, there were significant effects of angle (F_(3, 48)_ = 79.024, p<0.001, ε = 0.450), version (F_(1, 16)_ = 51.418, p<0.001) and the interaction between angle and version (F_(3, 48)_ = 24.636, p<0.001, ε = 0.518). However, no significant main effect of the factor hypoxic level or its interactions with the angle and version were observed. Similar to the error rate, the mean response time also showed an increment trend as angular deviation became more from upright (see Fig 2(B)). The response time with mirrored letters (M_mirrored_ = 882.940 ms, SE = 44.787 ms) was larger than that with normal letters (M_normal_ = 752.283 ms, SE = 31.513 ms). A further analysis of angle × version showed that angle simple effects of normal and mirrored letters were both significant (F_(3, 48)_ = 78.288, p<0.001, ε = 0.407; F_(3, 48)_ = 18.539, p<0.001, ε = 0.628, respectively). Both the linear trend (F_(1, 16)_ = 94.320, p<0.001) and quadratic trend (F_(1, 16)_ = 30.089, p<0.001) were fitted for normal letters, and there also existed a significant linear trend (F_(1, 16)_ = 22.901, p<0.001) and quadratic trend (F_(1, 16)_ = 5.581, p = 0.031) with mirrored letters. The paired contrast results revealed that the differences between angles were all significant (all the adjusted-p <0.005) with normal letters, whereas the only difference between 0° and 60° was not significant (p>0.1) with mirrored letters. Moreover, the version simple effect analysis at each angle indicated that the effect was not significant only at 180° (p = 0.571), while those at the other three angles were significant (all p<0.001).

### Rotation-related Negativity (RRN)

**Fig 3:**
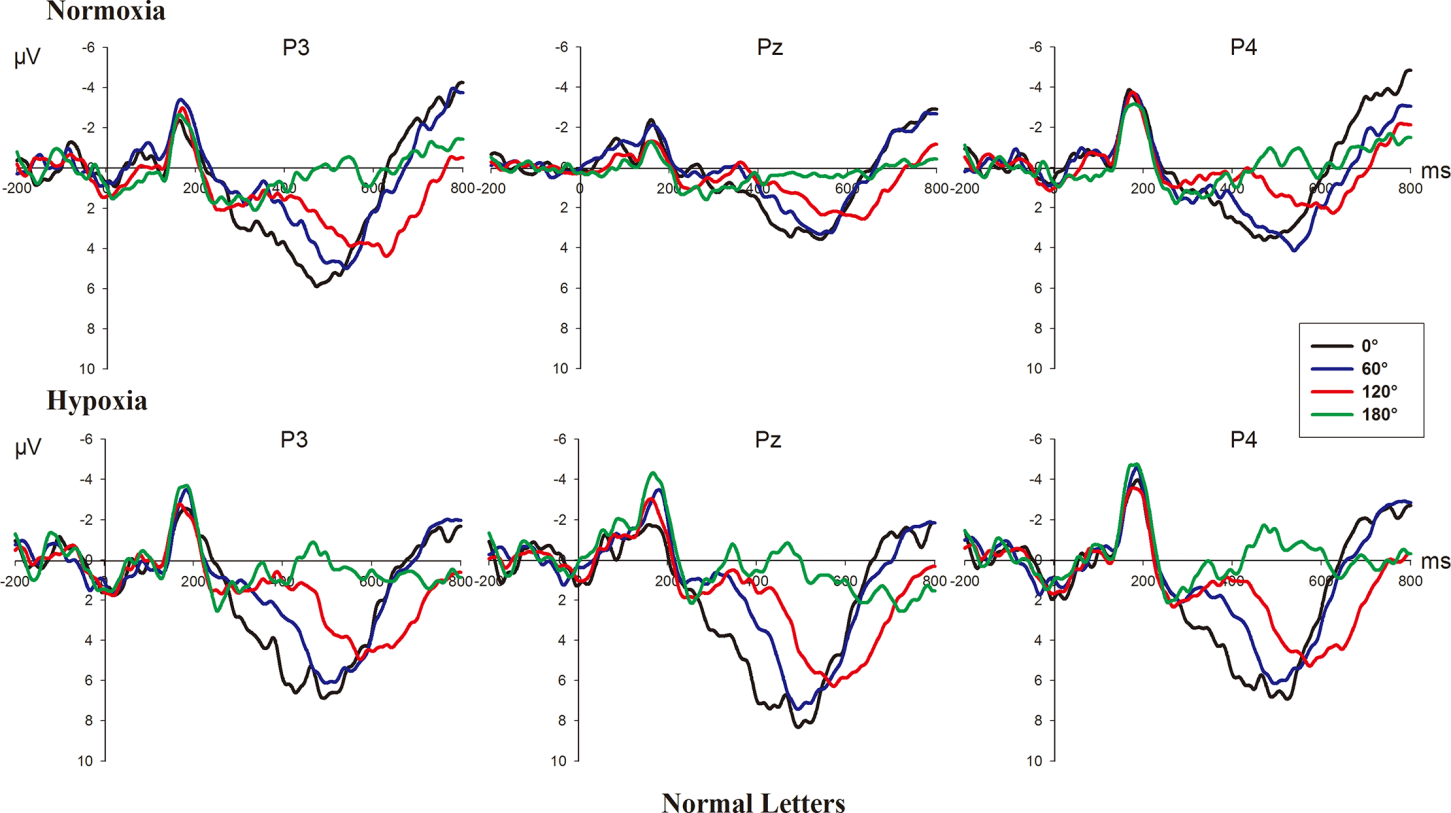
**Grand-average ERPs elicited by normal letters in each angle at P3, Pz, and P4 in normoxia (upper part) and hypoxia (bottom part) conditions.**

**Fig 4:**
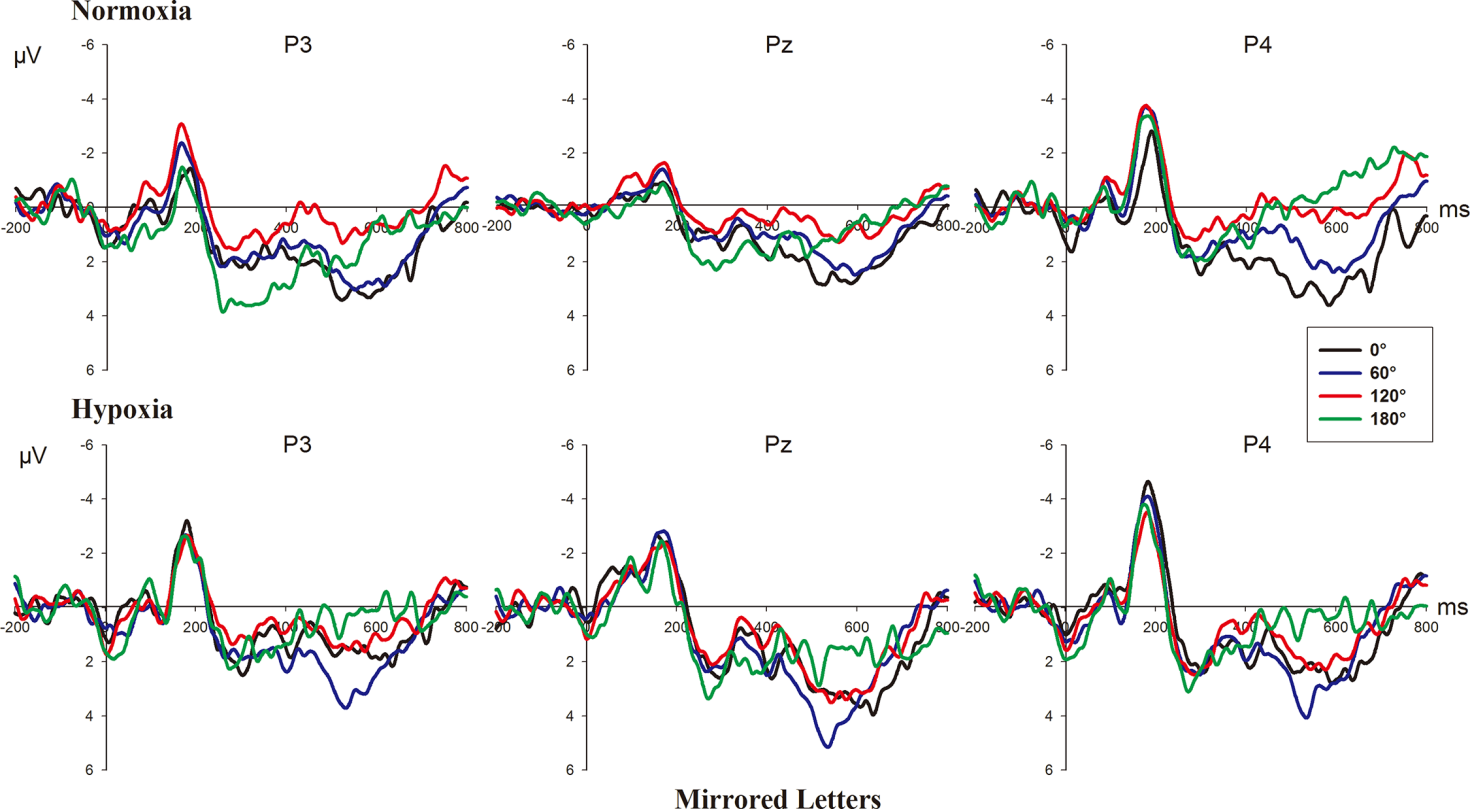
**Grand-average ERPs elicited by mirrored letters in each angle at P3, Pz, and P4 in normoxia (upper part) and hypoxia (bottom part) conditions.**

Figs 3 and 4 show the grand-average ERPs elicited by normal and mirrored letters in the four angles at the electrodes P3, Pz and P4 in the normoxia and hypoxia conditions.

#### RRN amplitude

The 2 (hypoxic level: normoxia vs. hypoxia) × 2 (version: normal vs. mirrored) × 4 (angle: 0°, 60°, 120° and 180°) × 3 (electrode: P3, Pz, and P4) repeated-measure ANOVA showed that there were significant main effects of hypoxic level (F_(1, 16)_ = 4.803, p = 0.044), version (F_(1, 16)_ = 24.345, p<0.001), and angle (F_(3, 48)_ = 26.605, p<0.001, ε = 0.561), but no significant effect of electrode was observed (F_(2, 32)_ = 1.673, p = 0.204). The mean amplitude of RRN in the normoxia condition was smaller than that of hypoxia (M_normoxia_ = 1.557, SE = 0.567; M_hypoxia_ = 2.323, SE = 0.590, respectively). Besides, the mean amplitude elicited by mirrored letters was smaller than that by the normal letters (M_mirrored_ = 1.291, SE = 0.566; M_normal_ = 2.588, SE = 0.569). With regard to the angle effect, the amplitude had a negative trend as the angular disparity from the upright position increased (see Figs 3 & 4). Moreover, the interactions of hypoxic level × version (F_(1, 16)_ = 7.306, p = 0.016), hypoxic level × angle (F_(3, 48)_ = 6.757, p = 0.004, ε = 0.661), hypoxic level × electrode (F_(2, 32)_ = 4.661, p = 0.017), version × angle (F_(3, 48)_ = 19.507, p<0.001), hypoxic level × version ×angle (F_(3, 48)_ = 7.591, p = 0.003, ε = 0.572) and hypoxic level × version × angle × electrode (F_(6, 96)_ = 3.975, p = 0.007, ε = 0.622) were all significant. Detailed results of simple effect analyses are given below.

Hypoxic effect: We further examined the hypoxic effect in the normal and mirrored letters conditions respectively. The results indicated the hypoxic effect was significant with the normal letters (F_(1, 16)_ = 12.184, p = 0.003), but not with the mirrored letters (F_(1, 16)_ = 0.377, p = 0.548). We also examined the hypoxic effects at the four angles, showing the significant effect at 0° (F_(1, 16)_ = 8.326, p = 0.011) and 60° (F_(1, 16)_ = 12.522, p = 0.003), but no significant effect at 120° (F_(1, 16)_ = 3.091, p = 0.098) and 180° (F_(1, 16)_ = 0.851, p = 0.37). Furthermore, in the upright position (0°) that can be used as the baseline for the other misorientation conditions [19], we found the hypoxic effect was significant only with upright-normal letters (F_(1, 16)_ = 25.513, p<0.001), but not with upright-mirrored letters (F_(1, 16)_ = 0.411, p = 0.530). Overall, the hypoxic effect was evident in the condition of small-rotated normal letters, with larger amplitude of RRN in the hypoxia condition than that in normoxia condition.

**Fig 5:**
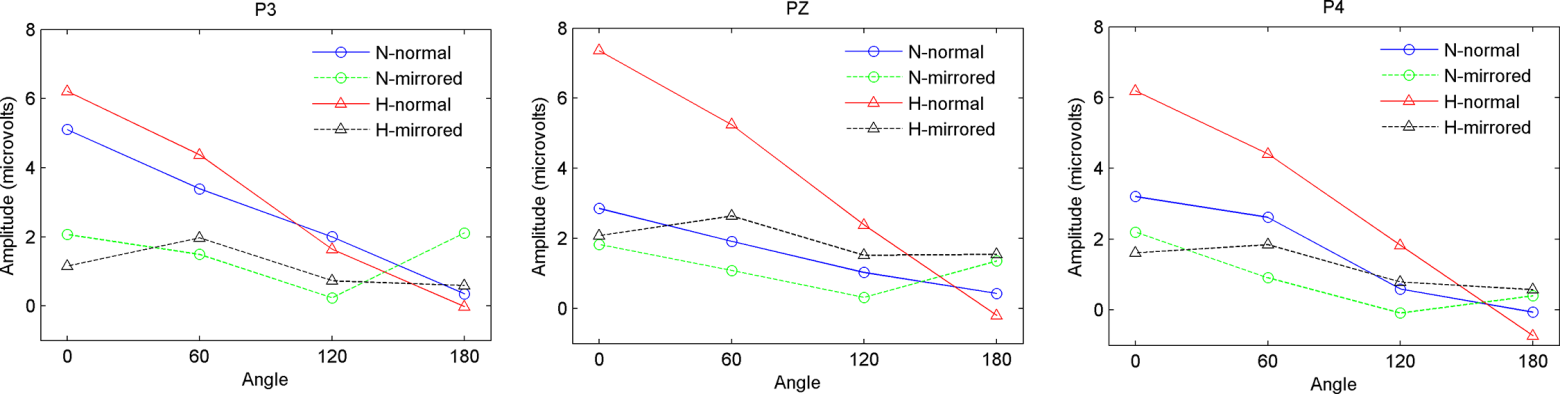
Mean amplitude of RRN in 400–500 ms time window as a function of angle at P3, Pz and P4. The blue solid line with circle denotes the normal letters in normoxia condition, the red solid line with triangle denotes the normal letters in hypoxia condition, the green dashed line with circle denotes the mirrored letters in normoxia condition, and the black dashed line with triangle denotes the mirrored letters in hypoxia condition.

Angle effect: Fig 5 shows the mean amplitude of RRN with the normal and mirrored letters as a function of angles at P3, Pz and P4 in the normoxia and hypoxia conditions. The simple effect analysis of angle was conducted in the normoxia and hypoxia conditions, which was significant in both conditions (F_(3, 48)_ = 11.743, p = 0.001, ε = 0.530; F_(3, 48)_ = 29.008, p<0.001, ε = 0.650, respectively). Since the interaction effect of version × angle was significant in both the normoxia and hypoxia conditions (F_(3, 48)_ = 7.249, p<0.001; F_(3, 48)_ = 21.469, p<0.001, respectively), we further analyzed the angle simple effect of the mirrored and normal letters at the two hypoxia levels, showing that the angle effect was not significant only with mirrored letters in the hypoxia condition (F_(3, 48)_ = 2.709, p = 0.055). There were not significant differences between all angles.

Version effect: The results of simple effects of version in the normoxia and hypoxia conditions revealed that a significant version effect existed in both the normoxia and hypoxia conditions (F_(1, 16)_ = 6.431, p = 0.022; F_(1, 16)_ = 29.432, p<0.001, respectively). With regard to the version effects at all angles in the normoxia and hypoxia conditions, the results revealed that the version effect reached significance at three angles (all p<0.05) except for the 180° (p = 0.092) in the normoxia condition, whereas the effect was significant at all the four angles in the hypoxia condition (all p<0.05).

**Fig 6:**
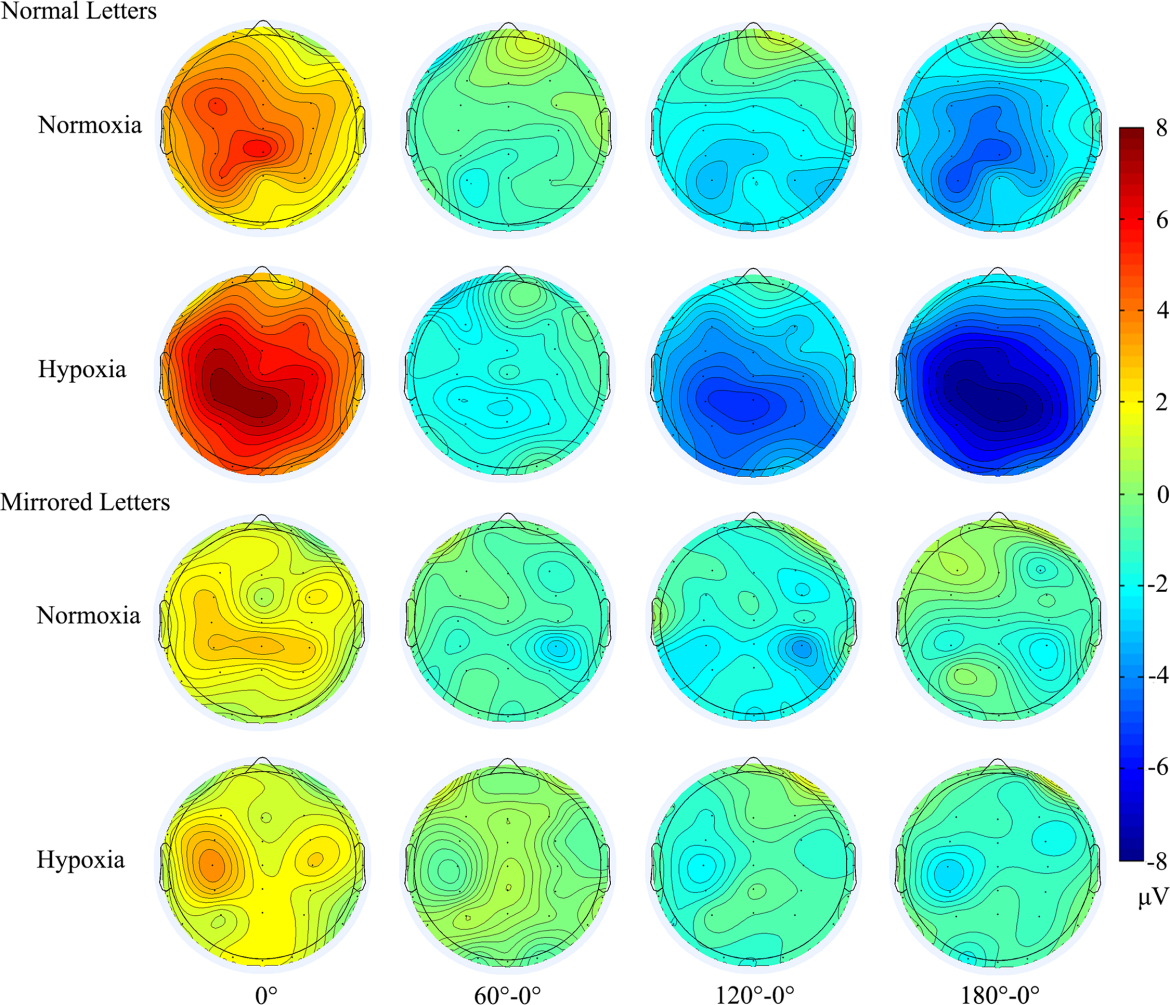
Spatial distribution of angle effect of normal and mirrored letters over all electrodes in normoxia and hypoxia conditions. From left to right are the voltage at 0°, voltage difference between 60°, 120°, 180° and 0° during the time window between 400 and 500 ms.

Electrode effect: Fig 6 shows voltage difference between 60°, 120°, 180° and 0° during the time window between 400 and 500 ms with normal and mirrored letters in different hypoxic level exposure and indicates that more bilateral brain is activated in the hypoxia condition. The simple effect analysis suggested electrode effect reached significance only in the normoxia condition (F_(2, 32)_ = 4.374, p = 0.021) but was not significant in the hypoxia condition (F_(2, 32)_ = 2.141, p = 0.153, ε = 0.678). The pairwise comparison revealed that there was a significant difference between the left and right parietal regions (p = 0.009, M_left_ = 2.094, SE = 0.599; M_right_ = 1.222, SE = 0.584). Additionally, version × angle × electrode was significant (F_(6, 96)_ = 4.942, p = 0.004, ε = 0.533) only in the normoxia condition but not in the hypoxia condition (F_(6, 96)_ = 0.959, p = 0.457, ε = 0.545). We further examined the electrode effect of the normal and mirrored letters respectively in the normoxia condition and observed the significant electrode effect only for normal letters (F_(2, 32)_ = 5.095, p = 0.012), while not for the mirrored letters (F_(2, 32)_ = 2.318, p = 0.115). The pairwise comparison revealed that there was a significant difference between the left and right parietal regions (p = 0.006, M_left_ = 2.712, SE = 0.611; M_right_ = 1.587, SE = 0.543).

#### RRN latency

The results of within-participant repeated-measure ANOVA showed that RRN latency with mirrored letters was significantly longer than that with normal letters (F_(1, 16)_ = 7.359, p = 0.015; M_normal_ = 439.787 ms, SE = 3.240; M_mirrored_ = 451.728 ms, SE = 3.947). Besides, the latency also revealed a significant angle effect (F_(3, 48)_ = 20.718, p<0.001, ε = 0.603). However, the main effects of hypoxic level and electrode were not significant (both p>0.1). The interaction effects of hypoxic level × angle (F_(3, 48)_ = 4.064, p = 0.029, ε = 0.636) and version × angle (F_(3, 48)_ = 7.212, p<0.001) were significant. We further examined the angle simple effect in the normoxia and hypoxia condition respectively, which was both significant (F_(3, 48)_ = 9.791, p = 0.001, ε = 0.563; F_(3, 48)_ = 21.204, p<0.001, respectively). The paired contrast results revealed that the differences between 180° and the other three angles were all significant in both normoxia (all adjusted-p <0.05) and hypoxia conditions (all adjusted-p <0.005), with the largest latency at 180°. Similarly, angle simple effects on the latency were significant with normal and mirrored letters (F_(3, 48)_ = 21.208, p<0.001, ε = 0.561; F_(3, 48)_ = 4.149, p = 0.011, respectively). The paired contrast results indicated that latency at 180° was significantly longer than those at the other three angles (all the adjusted-p <0.01) for normal letters, whereas no significant differences between angles were observed (all the adjusted-p> 0.05) for mirrored letters. Additionally, the version simple effect analysis at each angle indicated that the effects were significant at 60° (p = 0.001) and 120° (p = 0.023), while those at 0° (p = 0.107) and 180° (p = 0.485) were not significant.

## Discussion

The current study aimed to explore the modulation of brain activities underlying the mental rotation process during moderate and transient exposure to a hypoxia condition at a stimulated 5000 m altitude compared to the normoxia condition at sea level. An ERP experiment was conducted successively in normoxia and hypoxia conditions with the frequently used normal-mirrored letter discrimination task involving mental rotation. The behavioral results showed no overt behavioral alterations in the hypoxia condition, with no significant difference in response time or error rate between the normoxia and hypoxia conditions. We found the approximately classic linear functional relationship between response time and angular disparity from the upright position as previous studies [9, 16, 17, 20]. Besides, the difference in response time between normal and mirrored letters was also found, with the participants taking a longer time to judge mirrored letters [9, 19]. However, there were some differences between the behavior results and ERP results in reflecting the effect of hypoxia. First, the ERP results showed that the hypoxia indeed affect the mental rotation significantly, demonstrated by larger rotation-related negativity (RRN) amplitude in the hypoxia condition than that in the normoxia condition. However, this significant hypoxic effect occurred only in the condition of normal letters and disappeared with the letters at larger angles. Second, consistent with previous studies [9, 19], the significant angle effect, version effect and interaction effect of angle × version on the amplitude were observed in both the normoxia and hypoxia conditions, indicating that a neural response to mental rotation occurred in both these conditions. For normal letters, the angle effect became more evident in hypoxia condition compared to that in the normoxia condition (see Fig 3). However, for mirrored letters, angle effect was not as evident as normal letters in the normoxia condition and even became nonsignificant in the hypoxia condition (see Fig 4). With regard to the version effect, mirrored letters elicited smaller RRN amplitude than normal letters did at various angles except for the 180° in normoxia condition. While this normal-mirrored difference occurred at every angle in hypoxia condition. Finally, Fig 6 indicated that more bilateral brain activities in the hypoxia condition and significant hemispheric effect in the parietal area was observed only with normal letters in the normoxia condition. Regarding the results of RRN latency analysis, no significant hypoxic level effect was found. The above results indeed supported our hypotheses about the existence of hypoxic compensation effect as previous studies found [2, 13, 41, 42, 47] and revealed that moderate and transient hypoxic exposure might not induce impairment but enhancement in mental rotation performance with normal objects in the picture plane. On the contrary, this compensatory action could not be sufficient to enhance the mental rotation of mirrored objects.

Our behavioral results verified the angle effect and version effect found in previous studies [9, 10, 15, 19]. As shown in Fig 2, response time and error rate increased with larger angular deviation from the upright position for both normal and mirrored letters, indicating the participants rotated the mirrored letters as well as the normal letters. In accordance with previous studies [9, 19, 20, 22], the function between the angle and response time also showed a curved trend. According to the idea of coexistence of rotation and nonrotation strategies during mental rotation process [20, 22], the indifference of RT between between 0° and 60° with mirrored letters revealed that mental rotation might not be used in most trials of 60° rotation condition. Concerning the version effect, discriminating mirrored letters took longer time, implying the existence of an extra rotation out of the plane with mirrored letters [19]. Moreover, at large angle (180°), the RT of normal letters was not different from that of mirrored letters, which was in agreement with the finding reported by Núñez-Peña and Aznar-Casanova, who attributed this result to the increasing difficulty of large-angles rotation [9]. From the Fig 2, we can see that both the error rate and response time had the similar trends in the normoxia and hypoxia conditions. The absence of the differences in response time and error rate between hypoxia and normoxia conditions could be attributed to the compensation effect of hypoxia. The hypoxic level (5000m altitude) and exposure time (less than 15 minutes) we simulated in the experiment lay within the effective compensation range of cognitive functions [2, 42, 47]. Therefore, the compensatory actions of the participants to maintain the task performance were predictable. In several studies of visual-spatial abilities, there were also no obvious behavior alterations during exposure to hypoxia due to the compensation or adaptive mechanisms [13, 47]. In our study, this assumption was in agreement with their findings and was further verified by our ERP results.

In the current study, we used the rotation-related negativity as the electrophysiological measurement of mental rotation [27]. As shown in Fig 6, we can see that this component was mainly distributed over the parietal region within the time window between 400 and 500 ms, which was similar to previous findings [9, 17, 54]. As we know, the RRN is the overlap of a P300 and a slow negativity that goes more negative with greater angular disparity [16, 27]. The component elicited by upright-normal letters (0°) might have the same performance as P300 since participants did not rotate the letters in or out of the picture plane, which accounted for the negative deflection [16]. This condition has been suggested to be the baseline for the other misorientation conditions [19]. P300 is referred to as an index that reflects brain activity involved in mental representation [55] and stimulus discrimination and identification [3, 56]. Larger P300 indicates the more cognitive resources are allocated to the stimuli processing [57]. We found that the P300 amplitude of upright-normal letters was larger in the hypoxia condition than that in the normoxia condition, revealing that participants recruited more cognitive resources resulting from compensation mechanisms to maintain higher activation level against the deteriorative effect of hypoxia during the task. A prior study also found greater P300 activation induced by exposure to hypoxia during a visual categorical task, suggesting more effort was devoted by the participants to sustaining the task performance [47]. This compensation effect made the amplitude of rotation-related negativity larger and angle effect more evident with normal letters in the hypoxia condition demonstrated by the grand-average ERP in Fig 3 and topographic voltage difference in Fig 6. Besides, the enlarged P300 might partially resulted from a more bilateral brain activation pattern in the parietal region under the hypoxic exposure (see Fig 6). According to the analysis of RRN in P3, Pz and P4, there was a significant difference between the left and right sites in the parietal region in the normoxia condition for normal letters. However, it showed a more bilateral pattern for the mirrored letters. Previous studies proposed that the left parietal area was more activated than the right during rotation of two-dimensional stimuli, while the right parietal area was dominant for the three-dimensional stimuli [9, 35]. Since the mental rotation of mirrored letters involved both two- and three-dimensional rotations, both hemispheres would be recruited. In contrast, there was no difference between these two sites in the hypoxia condition for normal and mirrored letters. Notably, this bilateral activity in the hypoxia condition might indicate a compensation mechanism. According to Corballis [34], the right hemisphere is involved in the holistic mental rotation process, and the left hemisphere is involved in the analytic mental rotation process. Humans are expected to utilize adaptive strategies to ensure response accuracy, which in our study might be the application of both holistic and analytic processes. Consequently, we could see that the brain was more active in the hypoxia condition, resulting in larger P300. The latency of RRN under the hypoxia condition was not significantly different from that under the normoxia condition, which was also reported in previous studies, suggesting there might be a compensation effect of hypoxia [1, 47].

Based on behavioral results, the normal-mirrored difference in response time verified that the flip process existed with the mirrored letters. Furthermore, we also found significant version effect on the RRN amplitude and latency in both normoxia and hypoxia conditions, with the larger amplitude and shorter latency for normal letters than for mirrored letters, which also supported previous studies on the “flip” theory [9, 19, 54]. In accordance with previous studies, the angle effect on the amplitude and latency was found less evident with mirror letters than normal letters in a normal environment (see Fig 4) [9, 19]. The above phenomena were due to the flip rotation ERP effect, which further reduced the RRN amplitude of mirrored letters compared to the normal letters and meanwhile attenuated the planar-rotation ERP differences between angles. However, the angle effect on the amplitude nearly disappeared with the mirrored letters in the hypoxia condition. Besides, we found there was no significant difference in RRN amplitude between normoxia and hypoxia conditions when participants rotated the mirrored letters. These results implied the compensation effect of hypoxia was only effective for the normal letters and the mental rotation process of mirrored letters might be impaired a little during exposure to a hypoxic environment. This difference of hypoxic effect might be attributed to the extra flip rotation of mirrored letters. Previous studies have suggested compensation effect of hypoxia would decline when the task becomes difficult since difficult tasks would demand more cognitive resources to maintain the constant level of performance, which might be insufficient in the hypoxia condition [1, 2, 41]. Mental rotation of mirrored letters was regarded as a more difficult task due to the rotation both in and out of the picture plane, which requires additional mental workload compared to normal ones [10]. Besides, several studies have proposed that rotation out of the picture plane (i.e. in the three-dimensional space) is more effortful [24–26, 35]. Therefore, mental rotation of mirrored letters made the people devote more cognitive resources to achieve the task, and this extent of effort might exceed the effective range of compensation. The difficulty of flip rotation could be reflected in the lack of difference of P300 amplitude between the normoxia and hypoxia conditions for upright-mirrored letters in the current study. Specifically, the P300 amplitude elicited by upright-normal letters in hypoxia condition was larger than that in normoxia condition. However, for the upright-mirrored letters, the P300 amplitude in hypoxia condition decreased to be as equal as to the amplitude of upright-mirrored in the normoxia condition. Since the difference between upright-normal letters and upright-mirrored letter discrimination was whether flip rotation was involved or not, it seemed that this additional rotation would consume a lot of cognitive resources, indicating that flip rotation was difficult. Moreover, we found that the difference in RRN amplitude between normal and mirrored letters was not significant for large angle (180°) in the normoxia condition, which was in agreement with prior studies [9, 58]. It is because large-angles rotation requires heavy visuo-spatial demands, which reduces the advantage of normal letters over mirrored letters, and mental rotation in and out of the picture plane may occur in parallel for mirrored letters [9]. In contrast, the version effects on amplitude were significant for all angles in the hypoxia condition. We attributed this difference to the compensation, which maintains the advantage of normal letters and impairs the processing of mirrored letters. In addition, we also found the hypoxic effect disappeared with the larger-rotated letters relative to the upright and small-rotated letters. This result could be also due to the difficulty in mental rotating the letters at lager angle, which required higher cognitive load [9, 10, 27].

In sum, our work was the first study to uncover the underlying neural mechanisms of the hypoxic effect on spatial ability measured by mental rotation using the ERP method. We conclude that mental rotation is indeed influenced by hypoxia, even with short-term exposure. The compensation mechanism was found in the hypoxia condition and was sufficient to enhance the mental rotation of normal letters, while insufficient to maintain the performance of rotating the mirrored letters.

## Supporting Information

S1 Dataset Experimental data. (ZIP)
