# Correction: Different Effects of Hypoxia on Mental Rotation of Normal and Mirrored Letters: Evidence from the Rotation-Related Negativity

**DOI:** 10.1371/journal.pone.0213606

**Published:** 2019-03-05

**Authors:** Qingguo Ma, Linfeng Hu, Jiaojie Li, Yue Hu, Ling Xia, Xiaojian Chen, Wendong Hu

In the Funding section, the grant number from the funder National Project is listed incorrectly. The correct grant number is: AWS14J011.
